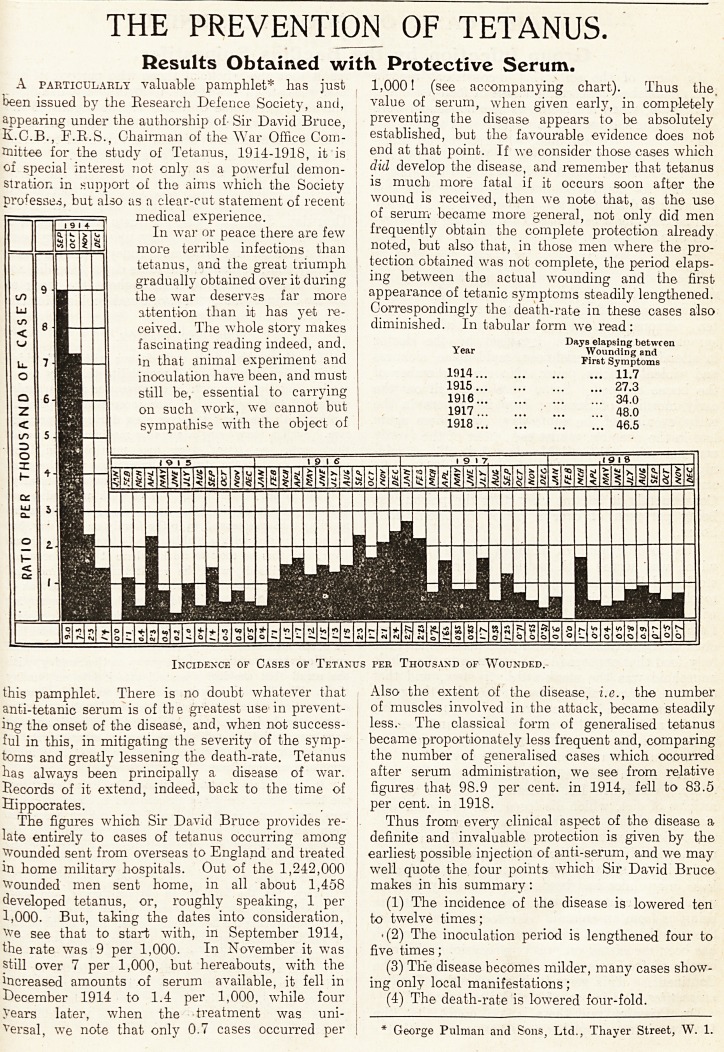# Results Obtained with Protective Serum

**Published:** 1920-08-21

**Authors:** 


					August 21, 1920. THE HOSPITAL. 523
THE PREVENTION OF TETANUS.
Results Obtained with Protective Serum.
A particularly valuable pamphlet* has just
been issued by the Research Defence Society, and,
appearing under the authorship of- Sir David Bruce,
K.C.B., F.B.S., Chairman of the War Office Com-
mittee for the study of Tetanus, 1914-1918, it is
of special interest not only as a powerful demon-
stration in support of the aims which the Society
professed, but also as a clear-cut statement of recent
medical experience.
In war or peace there are few
more terrible infections than
tetanus, and the great triumph
gradually obtained over it during
the war deserves far more
attention than it has yet re-
ceived. The whole story makes
fascinating reading indeed, and.
in that animal experiment and
inoculation have been, and must
still be, essential to carrying
on such work, we cannot but
sympathise with the object of
this pamphlet. There is no doubt whatever that
anti-tetanic serum is of tli e greatest use in prevent-
ing the onset of the disease, and, when not success-
ful in this, in mitigating the severity of the symp-
toms and greatly lessening the death-rate. Tetanus
has always been principally a disease of war.
Records of it extend, indeed, back to the time of
Hippocrates.
The figures which Sir David Bruce provides re-
late entirely to cases of tetanus occurring among
Wounded sent from overseas to England and treated
in home military hospitals. Out of the 1,242,000
Wounded men sent home, in all about 1,458
developed tetanus, or, roughly speaking, 1 per
1,000. But, taking the dates into consideration,
We see that to start with, in September 1914,
the rate was 9 per 1,000. In November it was
still over 7 per 1,000, but hereabouts, with the
increased amounts of serum available, it fell in
December 1914 to 1.4 per 1,000, while four
years later, when the treatment was uni-
versal, we note that only 0.7 cases occurred per
1,000! (see accompanying chart). Thus the.
value of serum, when given early, in completely
preventing the disease appears to be absolutely
established, but the favourable evidence does not
end at that point. If we consider those cases which
did develop the disease, and remember that tetanus
is much more fatal if it occurs soon after the
wound is received, then we note that, as the use
of serum became more general, not only did men
frequently obtain the complete protection already
noted, but also that, in those men where the pro-
tection obtained was not complete, the period elaps-
ing between the actual wounding and the first
appearance of tetanic symptoms steadily lengthened.
Correspondingly the death-rate in these cases also
diminished. In tabular form we read:
Year
1914...
1915...
1916...
1917...
1918 ...
Days elapsing between
Wounding and
First Symptoms
... 11.7
... 27.3
... 34.0
... 48.0
... 46.5
Also the extent of the disease, i.e., the number
of muscles involved in the attack, became steadily
less.- The classical form of generalised tetanus
became proportionately less frequent and, comparing
the number of generalised cases which occurred
after serum administration, we see from relative
figures that 98.9 per cent, in 1914, fell to 83.5
per cent, in 1918.
Thus from' every clinical aspect of the disease a
definite and invaluable protection is given by the
earliest possible injection of anti-serum, and we may
well quote the four points which Sir David Bruce
makes in his summary:
(1) The incidence of the disease is lowered ten
to twelve times ;
? (2) The inoculation period is lengthened four to
five times;
(3) The disease becomes milder, many cases show-
ing only local manifestations;
(4) The death-rate is lowered four-fold.
* George Pulman and Sons, Ltd., Thayer Street, W. 1.
THE PREVENTION OF TETANUS.
Results Obtained with Protective Serum.
A particularly valuable pamphlet* has just 1,000! (see accompanying chart). Thus the
been issued by the Research Defence Society, and, value of serum, when given early, in completely
appearing under the authorship of- Sir David Bruce, i preventing the disease appears to be absolutely
K.C.B., F.B.S., Chairman of the War Office Com- ' established, but the favourable evidence does not
ttiittee for the study of Tetanus, 1914-1918, it is I end at that point. If we consider those cases which
of special interest not- only as a powerful demon- J ^ develop the disease, and remember that tetanus
stration in support of the aims which the Society much more fatal if it occurs soon after the
professes, but also as a clear-cut statement of recent wound is received, then we note that, as the use
medical experience. serum1 became more general, not only did men
In war or peace there are few frequently obtain the complete protection already
more terrible infections than noted, but also that, in those men where the pro-
tetanus, and the great triumph tection obtained was not complete, the period elaps-
gradually obtained over it during ino between the actual wounding and the first
the war deserves far more appearance of tetanic symptoms steadily lengthened.
attention than it has yet re- Correspondingly the death-rate in these cases also
ceived. The whole story makes diminished. In tabular form we read:
fascinating reading indeed, and. ? Days elapsing between
. . . ? . ' J-ear Wounding and
m that animal experiment and First Symptoms
inoculation have been, and must 1914 11.7
still be, essential to carrying *^5 27.3
on such work, we cannot but 1917 .'.'. ... 48.0
sympathise with the object of 1918..,   46.5
Incidence of Cases of Tetanus per Thousand of Wounded.
this pamphlet. There is no doubt whatever that Also the extent of the disease, i.e., the number
anti-tetanic serum is of tlie greatest use in prevent- j of muscles involved in the attack, became steadily
ing the onset of the disease, and, when not success- : less. The classical form of generalised tetanus
ful in this, in mitigating the severity of the symp- became proportionately less frequent and, comparing
toms and greatly lessening the death-rate. Tetanus the number of generalised cases which occurred
has always been principally a disease of war
Records of it extend, indeed, back to the time of
Hippocrates.
The figures which Sir David Bruce provides re-
late entirely to cases of tetanus occurring among
Wounded sent from overseas to England and treated
in home military hospitals. Out of the 1,242,000
Wounded men sent home, in all about 1,458
after serum administration, we see from relative
figures that 98.9 per cent, in 1914, fell to 83.5
per cent, in 1918.
Thus from1 every clinical aspect of the disease a
definite and invaluable protection is given by the
earliest possible injection of anti-serum, and we may
well quote the four points which Sir David Bruce
makes in his summary
developed tetanus, or, roughly speaking, 1 per j (1) The incidence of the disease is lowered ten
1,000. But, taking the dates into consideration, i to twelve times;
We see that to start with, in September 1914, j .(2) The inoculation period is lengthened four to
the rate was 9 per 1,000. In November it was j five times;
still over 7 per 1,000, but hereabouts, with the j (3) The disease becomes milder, many cases show-
increased amounts of serum available, it fell in j ing only local manifestations;
December 1914 to 1.4 per 1,000, while four
years later, when the treatment was uni-
versal, we note that only 0.7 cases occurred per
(4) The death-rate is lowered four-fold.
George Pulman and Sons, Ltd., Thayer Street, W. 1.

				

## Figures and Tables

**Figure f1:**